# Supplementary material for: PremPRI: Predicting the Effects of Missense Mutations on Protein–RNA Interactions
Source: Int J Mol Sci. 2020 Aug 3;21(15):5560. doi: 10.3390/ijms21155560 (PMC7432928; doi:10.3390/ijms21155560)
Supplement: Supplementary file 1 [file ijms-21-05560-s001.pdf]

Supplementary Materials

PremPRI: Predicting the Effects of Missense Mutations on Protein-RNA Interactions

Ning Zhang[1], Haoyu Lu[1], Yuting Chen[1], Zefeng Zhu[1], Qing Yang[1], Shuqing Wang[1] and Minghui Li[1,*]

[1]Center for Systems Biology, Department of Bioinformatics, School of Biology and Basic Medical Sciences, Soochow University, Suzhou 215123, China

*corresponding author, minghui.li@suda.edu.cn

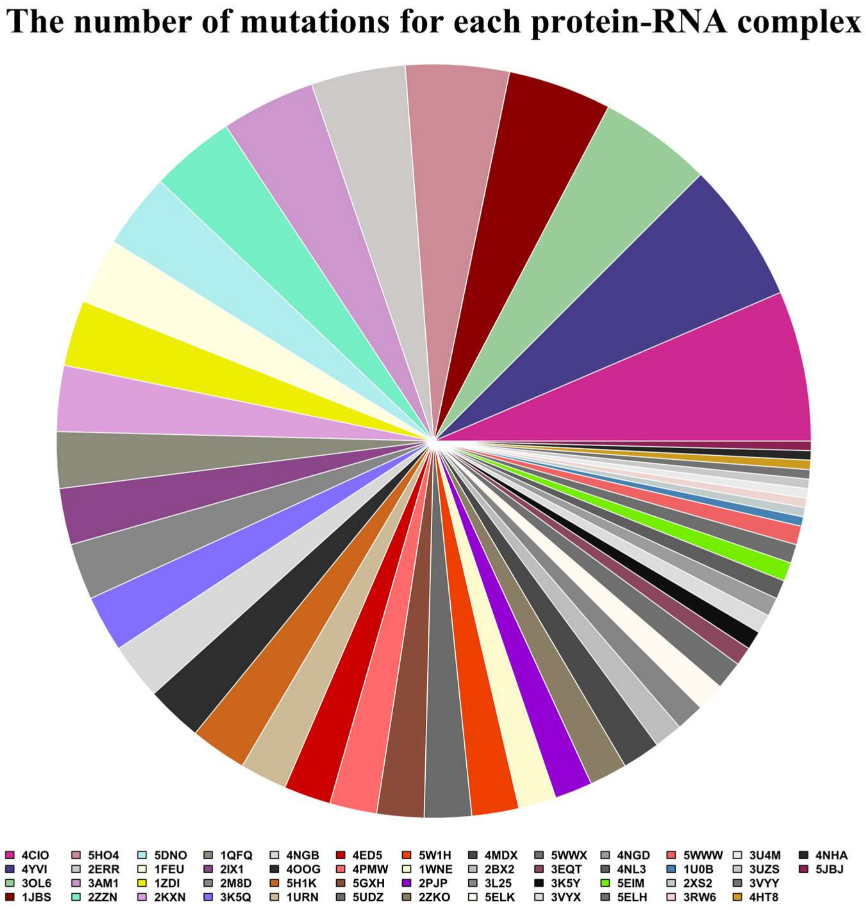

# of mutations	PDB ID of complex
16	4CIO
15	4YVI
12	3OL6
11	1JBS, 5HO4
10	2ERR, 3AM1
9	2ZZN
8	5DNO
7	1FEU, 1ZDI, 2KXN
6	1QFQ, 2IX1, 2M8D, 3K5Q, 4NGB, 4OOG, 5H1K
5	1URN, 4ED5, 4PMW, 5GXH, 5UDZ, 5W1H
4	1WNE, 2PJP, 2ZKO, 4MDX
3	2BX2, 3L25, 5ELK, 5WWX
2	3EQT, 3K5Y, 3VYX, 4NGD, 4NL3, 5EIM, 5ELH, 5WWW
1	1U0B, 2XS2, 3RW6, 3U4M, 3UZS, 3VYY, 4HT8, 4NHA, 5JBJ

Figure S1. The number of mutations for each protein-RNA complex in S248 dataset, which includes 248 mutations from 50 protein-RNA complexes.

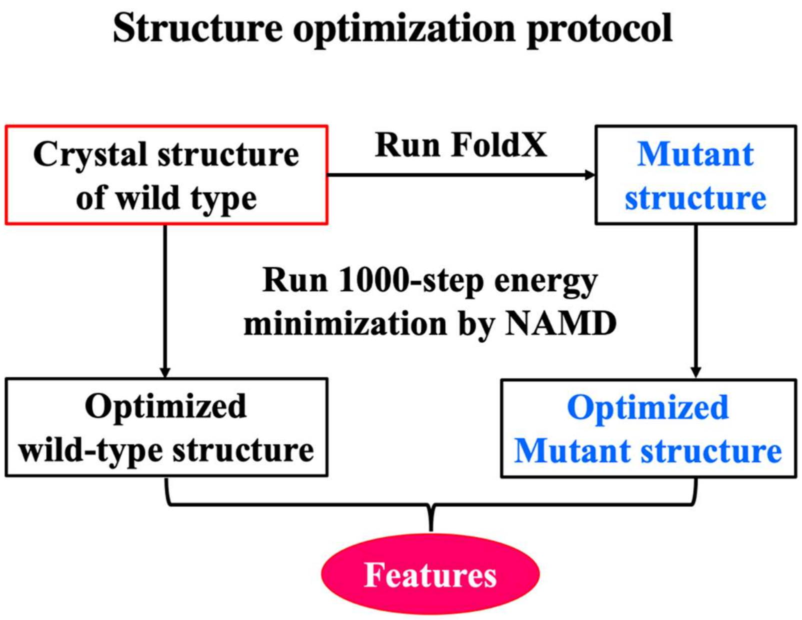

Figure S2. The flowchart of structure optimization protocol.

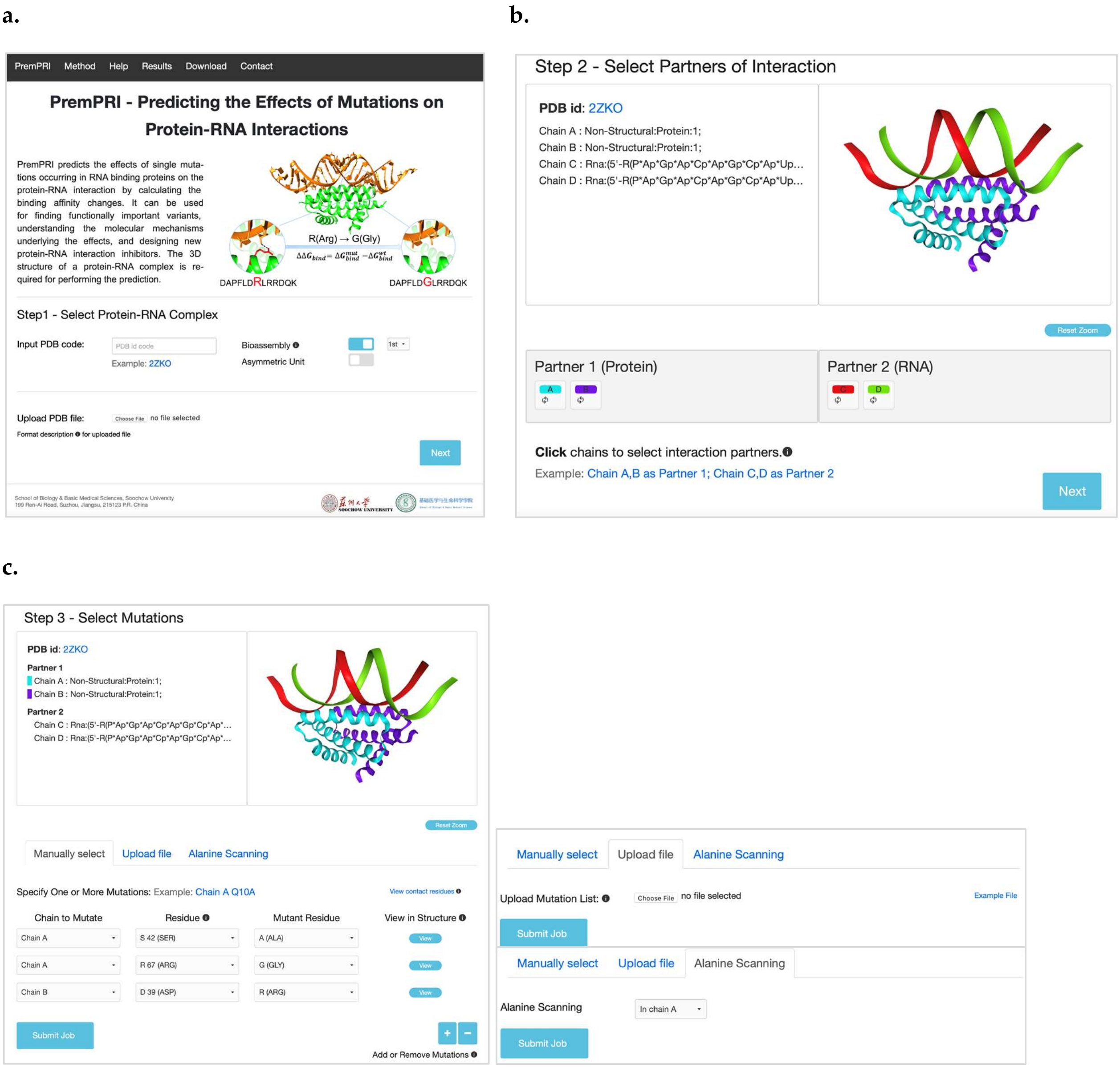

Figure S3. (a) The entry page of PremPRI server. (b) The second step for selecting interaction partners. (c) The third step for selecting mutations and three options are provided: "Specify One or More Mutations Manually", "Upload Mutation List" and "Alanine Scanning for Each Chain".

a.

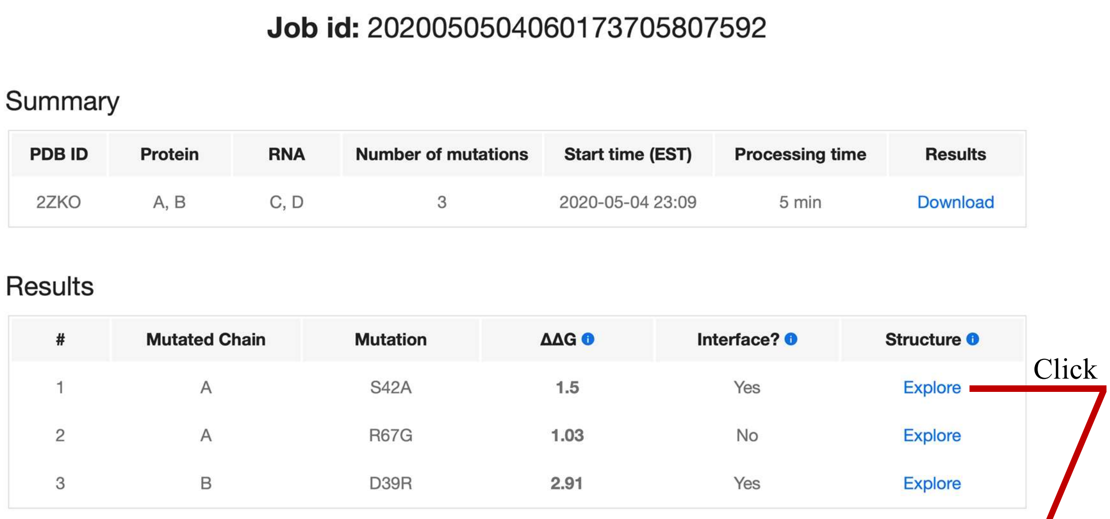

Job id: 20200505040601737058O7592

- Summary

PDB ID	Protein	RNA	Number of mutations	Start time (EST)	Processing time	Results
2ZKO	A, B	C, D	3	2020-05-04 23:09	5 min	Download

- Results

#	Mutated Chain	Mutation	ΔΔG	Interface?	Structure
1	A	S42A	1.5	Yes	Explore
2	A	R67G	1.03	No	Explore
3	B	D39R	2.91	Yes	Explore

b.

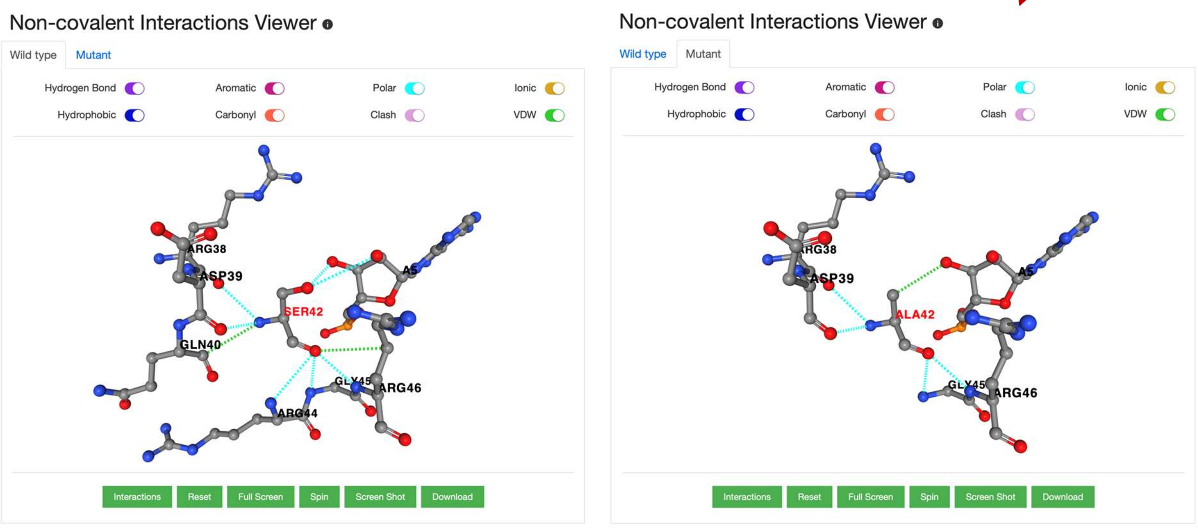

Figure S4. (a) The final results. "Processing time" refers to the running time of a job without counting the waiting time in the queue. (b) Interactive 3D viewer showing the non-covalent interactions between the mutated site in the NS1 protein of human influenza virus A (PDB ID: 2ZKO, mutation: S42A) and its adjacent residues/nucleotides in the wild-type (left) and mutant (right) complex respectively, generated by Arpeggio.

Table S1. Experimental datasets used for training methods of PremPRI, mCSM-NA and PrabHot.

Dataset	# of mutations	# of complexes	Description
S248	248	50	training set of PremPRI
S264	264(67)	33(5)	training set of mCSM-NA including mutations from both protein-DNA and -RNA complexes; bracket: the number of mutations from protein-RNA complexes
S151	151	32	training set of PrabHot, classification method
S16	16	2	overlap mutations between S248 and S264
S92	92	21	overlap mutations between S248 and S151

Different categories for mutations in S248			
**Category**	**# of mutations**	**# of complexes**	**Description**
Alanine-scanning	213	50	substitutions of residues into alanine
Non-alanine-scanning	35	13	substitutions of residues into non-alanine
Interface	154	45	mutations occur at protein-RNA binding interface
Non-interface	94	31	mutations do not occur at binding interface
Protein-ssRNA	122	24	mutations occur in protein-single stranded RNA complexes
Protein-dsRNA	126	26	mutations occur in protein-double stranded RNA complexes

Table S2. The p-value and importance of each feature in multiple linear regression scoring function of PremPRI. All Features have significant contribution to the quality of the model (p-value < 0.01, t-test). The features are ranked with respect to the importance.

Feature	P-value	Importance
$\Delta \boldsymbol{P}_{\boldsymbol{FWY}}$	1.40E-13	0.52
$\boldsymbol{N}_{\boldsymbol{inter}}$	7.24E-07	0.30
$\boldsymbol{Closeness}$	6.90E-06	0.30
$\boldsymbol{R}_{\boldsymbol{L/SA}}$	1.23E-05	0.29
$\Delta\Delta \boldsymbol{E}_{\boldsymbol{vdw.re}}$	1.18E-05	0.28
$\boldsymbol{P}_{\boldsymbol{coil}}$	2.07E-05	0.26
$\Delta\Delta \boldsymbol{E}_{\boldsymbol{elec}}$	1.49E-03	0.20
$\Delta \boldsymbol{SA}$	7.78E-04	0.19
$\Delta \boldsymbol{P}_{\boldsymbol{KR-DE}}$	5.13E-04	0.19
$\Delta \boldsymbol{OMH}$	3.27E-03	0.19
$\Delta\Delta \boldsymbol{E}_{\boldsymbol{vdw}}$	3.59E-03	0.15

Standardized coefficients are used for describing the importance.

Table S3. The performance using multiple linear regression (MLR), Random Forest (RF), Back Propagation Neural Network (BPNN), Support Vector Machine (SVM) and eXtreme Gradient Boosting (XGBoost) algorithms to build PremPRI model, respectively.

**Algorithm**	**Method**	**R**	**RMSE**	**Slope**
**MLR**	PremPRI	0.72	0.76	1.00
	PremPRI (CV3)	0.61	0.87	0.89
**RF**	PremPRI	0.70	0.79	1.21
	PremPRI (CV3)	0.46	0.98	1.15
**BPNN**	PremPRI	0.82	0.63	1.01
	PremPRI (CV3)	0.46	0.99	0.72
**SVM**	PremPRI	0.85	0.61	1.25
	PremPRI (CV3)	0.42	1.00	0.89
**XGBoost**	PremPRI	0.99	0.14	1.09
	PremPRI (CV3)	0.40	1.00	0.95

PremPRI: trained and tested on S248 dataset; PremPRI (CV3): leave-one-complex-out validation results.

R: Pearson correlation coefficient. RMSE (kcal mol^{-1}): root-mean-square error. Slope: the slope of the regression line between experimental and predicted ΔΔ[illegible] values. All presented correlation coefficients are statistically significantly different from zero (p-value << 0.01, t-test).

Table S4. Variance inflation factor (VIF) of each feature in PremPRI model. The features are ranked with respect to the VIF. The VIF of each feature is less than three, indicating low collinear relationships among 11 independent variables.

Feature	VIF
$\Delta \boldsymbol{P}_{FWY}$	2.20
$\Delta \boldsymbol{OMH}$	2.13
$\boldsymbol{R}_{L/SA}$	2.12
$\boldsymbol{Closeness}$	2.07
$\Delta\Delta \boldsymbol{E}_{vdw.re}$	1.97
$\Delta\Delta \boldsymbol{E}_{elec}$	1.85
$\boldsymbol{P}_{coil}$	1.81
$\boldsymbol{N}_{inter}$	1.69
$\Delta \boldsymbol{SA}$	1.61
$\Delta \boldsymbol{P}_{KR-DE}$	1.41
$\Delta\Delta \boldsymbol{E}_{vdw}$	1.25

Table S5. PremPRI performance for different categories of mutations.

Mutation category	Method	R	RMSE	Slope
**Alanine-scanning**	PremPRI	0.71	0.74	1.01
	PremPRI (CV3)	0.61	0.83	0.89
**Non-alanine-scanning**	PremPRI	0.78	0.85	0.98
	PremPRI (CV3)	0.60	1.08	0.90
**Interface**	PremPRI	0.75	0.78	1.05
	PremPRI (CV3)	0.62	0.93	0.93
**Non-interface**	PremPRI	0.65	0.71	0.86
	PremPRI (CV3)	0.58	0.77	0.76
**Protein-ssRNA**	PremPRI	0.76	0.82	1.08
	PremPRI (CV3)	0.61	0.98	0.99
**Protein-dsRNA**	PremPRI	0.64	0.69	0.84
	PremPRI (CV3)	0.59	0.75	0.74

PremPRI: trained and tested on S248 dataset; PremPRI (CV3): leave-one-complex-out validation results.

R: Pearson correlation coefficient. RMSE (kcal mol^{-1}): root-mean-square error. Slope: the slope of the regression line between experimental and predicted $\Delta\Delta G$ values. All presented correlation coefficients are statistically significantly different from zero (p-value << 0.01, t-test).

Table S6. Average weighting coefficient and the corresponding standard deviation (in bracket) for each feature in three types of cross-validation (CV1-CV3). The weighting coefficient in the PremPRI model is presented for the comparison. The features are ranked with respect to the absolute value of weighting coefficient in the PremPRI model.

Feature	CV1	CV2	CV3	PremPRI
$\Delta \boldsymbol{P}_{FWY}$	-218.13(31.43)	-216.40(15.44)	-215.51(9.57)	-216.23
$\boldsymbol{R}_{L/SA}$	83.93(17.89)	83.01(10.69)	83.53(4.26)	83.51
$\Delta \boldsymbol{P}_{KR-DE}$	-31.65(16.34)	-32.70(7.45)	-33.34(3.73)	-33.48
$\boldsymbol{Closeness}$	5.59(1.33)	5.66(0.77)	5.77(0.31)	5.77
$\boldsymbol{P}_{coil}$	-5.95(1.68)	-5.71(0.81)	-5.66(0.44)	-5.67
$\Delta \boldsymbol{OMH}$	0.21(0.06)	0.21(0.03)	0.21(1.42E-02)	0.21
$\Delta\Delta \boldsymbol{E}_{vdw.re}$	-0.11(2.57E-02)	-0.11(1.19E-02)	-0.11(5.31E-03)	-0.11
$\Delta\Delta \boldsymbol{E}_{vdw}$	0.02(6.90E-03)	0.02(3.81E-03)	0.02(1.20E-03)	0.02
$\boldsymbol{N}_{inter}$	-9.55E-03(2.01E-03)	-9.27E-03(7.24E-04)	-9.21E-03(4.41E-04)	-9.20E-03
$\Delta \boldsymbol{SA}$	5.47E-03(1.70E-03)	5.56E-03(9.66E-04)	5.52E-03(3.34E-04)	5.54E-03
$\Delta\Delta \boldsymbol{E}_{elec}$	1.07E-03(3.66E-04)	1.16E-03(1.72E-04)	1.14E-03(3.90E-05)	1.14E-03
**Intercept**	-0.33(0.54)	-0.39(0.30)	-0.41(0.14)	-0.41

Table S7. Comparison of methods' performances on three mutations from TthL1–RNA complex. $\Delta\Delta G_{exp}$ and $\Delta\Delta G_{pred}$ are experimentally determined and predicted binding affinity change (in kcal mol^{-1}), respectively.

Mutation	$\Delta\Delta G_{exp}$	PremPRI	mCSM-NA	FoldX	PrabHot
T217A	2.49	1.32	-1.18	-1.22	hotspot
T217V	3.61	1.87	1.20	0.12	hotspot
M218L	6.58	1.67	1.59	0.13	hotspot
G219V	5.35	1.94	-1.53	0.24	non-hotspot

Our training dataset of S248 includes one mutation of T217A from this complex, which was excluded from the training dataset when testing on this case.
